# Targeting exertional breathlessness to improve physical activity: the role of primary care

**DOI:** 10.1038/s41533-021-00254-8

**Published:** 2021-09-09

**Authors:** Miguel Román-Rodríguez, Janwillem W. H. Kocks

**Affiliations:** 1 Son Pisà Primary Health Care Centre, Balearic Health System, Mallorca, Spain; 2 Primary Care Chronic Respiratory Research Unit, Instituto de Investigación Sanitaria de las Islas Baleares (IdISBa), Mallorca, Spain; 3 General Practitioners Research Institute, Groningen, The Netherlands; 4 Groningen Research Institute for Asthma and COPD (GRIAC), University Medical Center Groningen, University of Groningen, Groningen, The Netherlands; 5 Observational and Pragmatic Research Institute, Singapore, Singapore

**Keywords:** Lifestyle modification, Patient education, Quality of life, Chronic obstructive pulmonary disease, Rehabilitation

## Abstract

Primary care physicians (PCPs) play a crucial role in the diagnosis and management of chronic obstructive pulmonary disease (COPD). By working together with patients to target exertional breathlessness and increase physical activity, PCPs have an important role to play, early in the disease course, in improving patient outcomes in both the short and long term. In this article, we consider how physical activity affects disease progression from the PCP perspective. We discuss the role of pharmacological therapy, the importance of an holistic approach and the role of PCPs in assessing and promoting physical activity. The complexity and heterogeneity of COPD make it a challenging disease to treat. Patients’ avoidance of activity, and subsequent decline in capacity to perform it, further impacts the management of the disease. Improving patient tolerance of physical activity, increasing participation in daily activities and helping patients to remain active are clear goals of COPD management. These may require an holistic approach to management, including pulmonary rehabilitation and psychological programmes in parallel with bronchodilation therapy, in order to address both physiological and behavioural factors. PCPs have an important role to optimise therapy, set goals and communicate the importance of maintaining physical activity to their patients. In addition, optimal treatment that addresses activity-related breathlessness can help prevent the downward spiral of inactivity and get patients moving again, to improve their overall health and long-term prognosis.

## Introduction

Chronic obstructive pulmonary disease (COPD) is a chronic disease and a major health problem worldwide^1^. The hallmark of COPD is airflow limitation, and patients suffer with persistent respiratory symptoms, such as cough, sputum production and dyspnoea (also referred to as breathlessness^1^). This can lead to a reduction in physical activity in the early stages of COPD^2^.

Primary care physicians (PCPs), who play a crucial role in the diagnosis and management of COPD, face a number of challenges in the diagnosis of the disease. These include a lack of specific symptoms, the failure of patients to report symptoms, the presence of multiple chronic conditions, inadequate training of physicians, nurse practitioners and physician assistants, and a lack of access to spirometry^3^. Lack of patient awareness regarding the disease also contributes to delayed diagnosis; the early symptoms of COPD, such as breathlessness and reduced exercise tolerance, are often attributed by patients to aging^4,5^.

PCPs experience several challenges with patients who already have a confirmed diagnosis of COPD, including (a) non-adherence to prescribed therapies, which is common in patients with COPD due to the chronic nature of the condition and the use of multiple medications^6^; (b) inhaler selection, which can be challenging given the wide range of different inhalation devices available^7^; (c) poor inhaler technique, which is often responsible for sub-optimal treatment of COPD, making device training essential^8,9^; (d) different perceptions between patients and physicians as to how COPD affects daily living; (e) different perceptions between younger and older patients on the impact of the disease on their quality of life^10^; and (f) challenges with smoking cessation^1^.

One of the most common symptoms of COPD is breathlessness. Breathlessness is distressing and debilitating and has a severe impact on all aspects of patients’ lives, preventing them from participating in certain daily activities^11,12^. Reducing physical activity is a natural and socially accepted way of avoiding symptoms, and there are several factors that affect engagement in physical activity. These include low motivation, correlated with fear of breathlessness and comorbidity^13^, and sociodemographic factors, such as age, race and social support^14^. Limiting physical activity due to breathlessness, sometimes referred to as “exertional breathlessness”, leads to a downward spiral of muscle wastage and further reductions in activity, ultimately leading to poor patient outcomes such as reduced quality of life and premature mortality^15–17^. Exacerbations are also associated with worse breathlessness, a reduction in exercise capacity and muscle weakness^18^.

By working together with patients to target exertional breathlessness and increase physical activity, PCPs have an important role to play (especially early in the disease course) in improving patient outcomes in both the short and long term. Agreement between PCPs and patients on the importance of physical activity may improve management of COPD^19^. In this educational narrative review, we consider the PCP perspective on physical activity and how it affects disease progression. We discuss the role of pharmacological therapy, the importance of an holistic approach (including pulmonary rehabilitation and psychological programmes) and the role of PCPs in managing physical activity.

## Physical activity and exercise capacity: predictors of poor outcomes in COPD

**Fig. 1:**
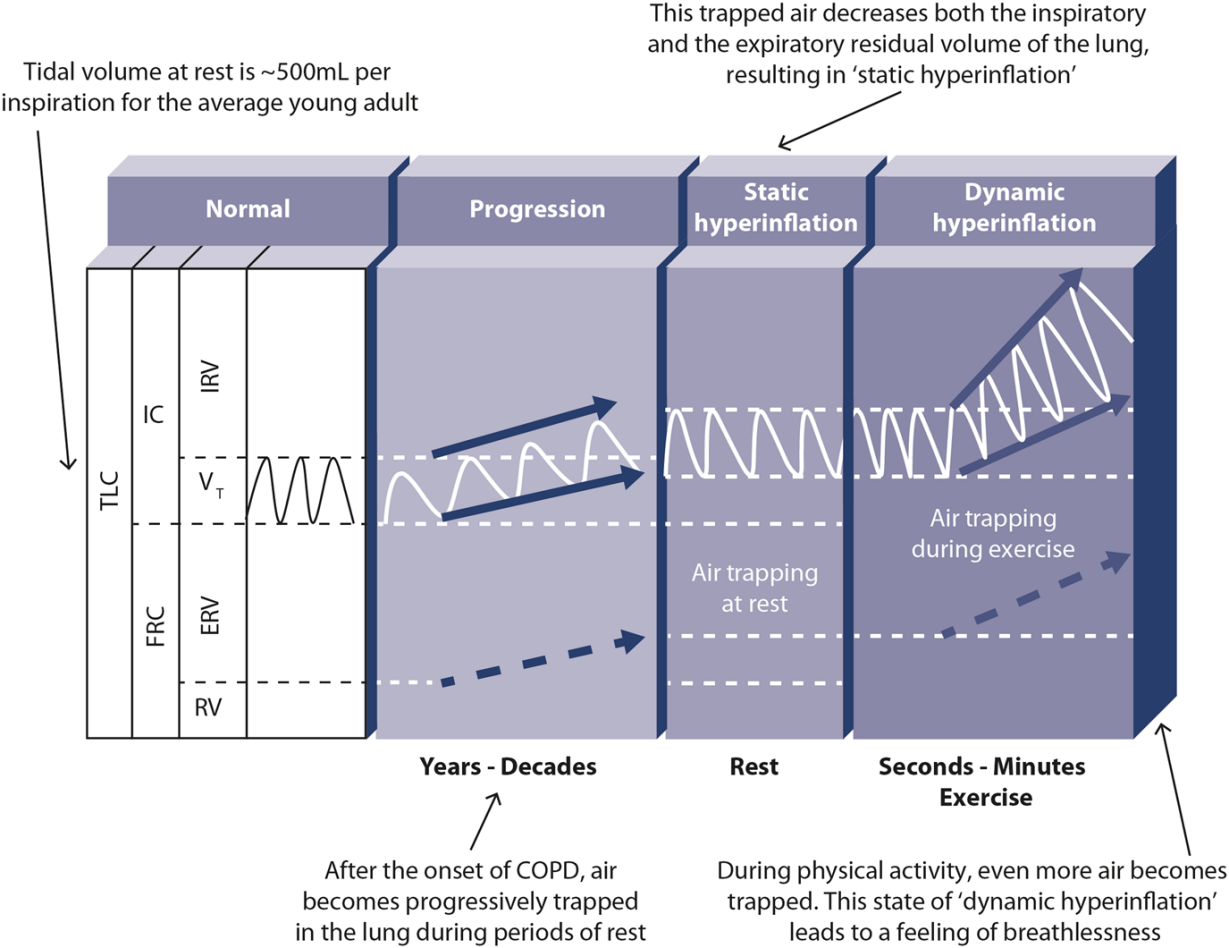
Relationship between hyperinflation and breathlessness in patients with COPD. ERV expiratory reserve volume, FRC functional residual capacity, IC inspiratory capacity, IRV inspiratory reserve volume, RV residual volume, TLC total lung capacity, *V*_T_ tidal volume.

Individuals with COPD frequently limit their activity because of activity-related breathlessness, often early in the disease course^2,20–23^. During physical activity, as a patient’s breathing rate and tidal volume increases, the time for expiration shortens and the degree of gas trapping worsens. This is typically assessed by demonstrating a reduction in inspiratory capacity during exercise. This phenomenon is called “dynamic hyperinflation” and is a key cause of the feeling of breathlessness during physical activity^24^. This process begins in the early stages of COPD and worsens with disease progression^12^. As such, sedentary behaviour occurs across the spectrum of COPD severity, from mild to very severe, and has negative implications for patients’ prognosis^2,25^ (Fig. 1).

Physical activity refers to the range of bodily movements that a patient undertakes as part of their normal daily routine. Low physical activity is an independent predictor for exacerbations, hospital admissions and mortality in patients with COPD^26–29^. In fact, it is one of the strongest predictors of all-cause and disease-specific mortality in COPD^30,31^. In addition, maintaining physical activity has been noted to decrease depression and anxiety over time^32^. Exercise capacity is a related concept that refers to the maximum extent of physical movement that a patient is capable of undertaking, but it does not necessarily correlate with levels of physical activity. A reduction in exercise capacity is also a predictor of poor outcomes, such as mortality and decreased quality of life, in patients with COPD^33,34^.

Sustaining physical activity is an important component of preventative medicine. Even if the progression of COPD prevents patients from increasing their level of physical activity, preserving exercise capacity allows them to maintain activity levels for longer before they become limited by exertional breathlessness. Within clinical trials, symptoms and exercise capacity are often measured, but there are less data on physical activity for evaluation. It is key that PCPs are aware of their patients’ health status and level of deconditioning before taking measures to improve physical activity. To facilitate this, assessment frameworks can help to profile patients and guide their referral to exercise-based care programmes—including ones that involve physiotherapy or more specialised pulmonary rehabilitation^35^.

## Tools to measure physical activity and exercise capacity

Regular measurement of physical activity in patients with COPD by PCPs, both initially and at follow-up, is vital in helping patient adherence to physical activity programmes^36^. Many different tools are available to PCPs to evaluate the functional status of their patients, including their ability to sustain physical activity and their overall exercise capacity. Some physical activity questionnaires, such as the International Physical Activity Questionnaire—Short Form (IPAQ-SF), are non-specific to COPD. The accuracy of these instruments in assessing physical activity is variable; for example, a review of studies that used the IPAQ-SF questionnaire showed only weak evidence as a predictor of relative or absolute physical activity^37^. The Physical Activity Scale for the Elderly is another widely used questionnaire, which has been shown to be a valid measure of physical activity, health and physical function in elderly patients in epidemiology studies^38^. Further activity questionnaires available include the Stanford Seven-day Physical Activity Recall Questionnaire^39^ and the Yale Physical Activity Survey^40^; however, these questionnaires may be more appropriate in a research environment than a primary care setting.

Specific tools to measure physical activity in COPD have also been produced, such as the London Chest Activity of Daily Living scale^41^ and the PROactive instrument^42^. The London Chest Activity of Daily Living scale^41^ is a questionnaire that assesses the impact of dyspnoea on activities of daily living in patients with severe COPD. However, it may be less practical for PCPs in their day-to-day practice due to the time it takes to complete and the difficulty of evaluating its results. Activity monitoring using wearable devices such as the PROactive instrument^39^ is not common in the primary care setting, but this may change with the evolution of digital healthcare. In addition, the Spanish Physical Activity Questionnaire in COPD has been specifically designed for an easy measurement of physical activity in patients with COPD during daily clinical practice^43^.

PCPs may also refer patients to a physiotherapist for assessment of functional impairments and determination of the most appropriate intervention to improve physical activity and exercise tolerance^44^. Physical activity can also be assessed objectively using pedometers. Pedometers can provide feedback on daily activities and allow patients to track their own progress^45^.

More practical approaches to measure functional performance in primary care include the British Medical Research Council dyspnoea questionnaire and the functional status domain of the Clinical COPD Questionnaire (CCQ)^46^. The British Medical Research Council dyspnoea questionnaire is a simple tool that grades the effect of breathlessness on daily activities^47^. However, its responsiveness to change is limited. The CCQ is another reliable yet quick and easy-to-use tool, consisting of ten items that cover symptoms and the functional and mental state of the patient^48^. The CCQ has been used in assessing interventions such as pulmonary rehabilitation, and is responsive to change, making it suitable for longitudinal use^48^.

Overall, although there are a number of tools available to measure physical activity and exercise capacity in patients with COPD, simple objective measures and quick and reliable questionnaires to capture patients’ symptoms and functional state are the most suitable for use in primary care.

## The role of bronchodilators on physical activity in COPD: review of data on activity-related endpoints with long-acting bronchodilators

One of the cornerstones of COPD management is effective pharmacological treatment to reduce symptoms and exacerbations as well as improving exercise tolerance and health status^1^. A systematic review and meta-analysis of 22 studies found that long-acting bronchodilators (either as monotherapy or in combination) increase exercise capacity in patients with COPD^49^. The authors of the review noted that this appears to be mainly due to an increase in inspiratory capacity rather than a modification of dynamic hyperinflation during exercise^49^. Several studies have demonstrated the benefits of long-acting muscarinic antagonist (LAMA)/long-acting β_2_-agonist (LABA) dual therapy in improving lung function and health-related quality of life, and in reducing symptoms, in patients with COPD^50^. Umeclidinium/vilanterol has been shown to reduce breathlessness versus placebo and its monocomponents^51^. Although randomised trial data evaluating exercise endurance showed that umeclidinium/vilanterol improves measures of lung function, hyperinflation and health status versus placebo, the effects on exercise endurance were variable^52,53^. Glycopyrronium/indacaterol has been shown to reduce hyperinflation and improve physical activity levels versus placebo, including increasing peak inspiratory capacity, activity-related energy expenditure and average number of steps per day^54^. It has also been demonstrated that aclidinium/formoterol improves breathlessness and overall night-time and early-morning symptom severity versus placebo and monocomponents^55^. Clinical trials have demonstrated that tiotropium/olodaterol improves breathlessness^56,57^ (including during exercise tests^58^), lung hyperinflation^59^ and inspiratory capacity^58,60^ compared with its monocomponents, and activity-related breathlessness and breathing discomfort versus placebo^60^. This combination has also been shown to increase exercise capacity during walking and cycling tests^60,61^ versus placebo and improve physical activity in treatment-naive patients, as indicated by reductions in breathlessness and in the amount of time spent in a sedentary position^59,62–65^. Notably, in a multicentre, multinational clinical trial, improvements from baseline in exercise endurance time for both cycling (19%) and walking (20%) were observed in patients with COPD following 6 weeks of treatment with tiotropium/olodaterol^66^. Overall, in clinical trials, long-acting bronchodilators are associated with improvements in exercise-related endpoints, with dual bronchodilation generally better than monotherapy.

## Real-world studies

In addition, several real-world studies suggest that bronchodilators have a beneficial effect on physical activity. In terms of monotherapy, the ON-AIR real-world evidence study evaluated the effects of the LAMA aclidinium bromide on quality of life, symptom severity and daily activity impairment in patients with COPD^67^. Aclidinium therapy improved quality of life, as demonstrated by reductions in mean COPD Assessment Test™ score, and reduced the severity of night-time and early-morning symptoms^67^. At least moderate impairment in performance of daily activities due to COPD symptoms was reported by 59.5% of patients at enrolment, improving to 38.7% after 12 weeks of aclidinium treatment^67^. Tiotropium improves physical functioning, with 61.5% of patients achieving an improvement of ≥10 points in the Physical Function subdomain after 6 weeks of treatment^68^. Furthermore, dual bronchodilation with tiotropium/olodaterol has been shown to improve physical function in patients with COPD^62–64^. This combination has also been shown to improve patients’ general condition and ability to manage their daily routines within 6 weeks of treatment^63,64^. In addition, a further open-label, non-interventional study demonstrated improvements in clinical health status, measured using the CCQ, in patients with COPD taking tiotropium/olodaterol in routine clinical practice^69^.

## The main role of bronchodilators on physical activity in COPD: what do the guidelines say?

Long-acting bronchodilators are central to the treatment of COPD. The Global Initiative for Chronic Obstructive Lung Disease (GOLD) 2020 strategy report recommends starting with a LAMA or LABA as first-line maintenance therapy for most patients with COPD^1^. For highly symptomatic patients, LAMA plus LABA dual therapy should be considered at initiation of pharmacological treatment, whereas LABA plus inhaled corticosteroid (ICS) should be considered in patients with previous exacerbations (≥2 moderate exacerbations or ≥1 exacerbation leading to hospitalisation) and with blood eosinophil levels ≥300 cells/µL^1^. Indeed, many patients do remain symptomatic on LABA or LAMA monotherapy^70^. In line with this, the American Thoracic Society (ATS) strongly recommends LAMA plus LABA dual therapy over LABA or LAMA monotherapy in patients who experience breathlessness or exercise intolerance, based on improved critical outcomes with dual therapy^71^. Similarly, the United Kingdom’s National Institute for Health and Care Excellence (NICE) guidelines recommend (a) LAMA plus LABA (no ICS) for patients without asthmatic features/features suggesting steroid responsiveness who remain breathless or have exacerbations despite smoking cessation treatment, (b) optimised non-pharmacological management and (c) use of a short-acting bronchodilator^72^.

The GOLD, ATS and NICE recommendations^1,71,72^ reflect the growing awareness of the importance to treat patients effectively from the initiation of treatment while also individualising COPD therapy according to the needs of the patient. Considering this guidance, PCPs should be aiming to optimise both non-pharmacological management and pharmacological therapy at an early stage of the disease^1,71–73^, which should increase physical activity and lead to improved prognosis for patients with COPD.

## Importance of an holistic approach, including pharmacological treatment, pulmonary rehabilitation and psychological programmes

**Fig. 2:**
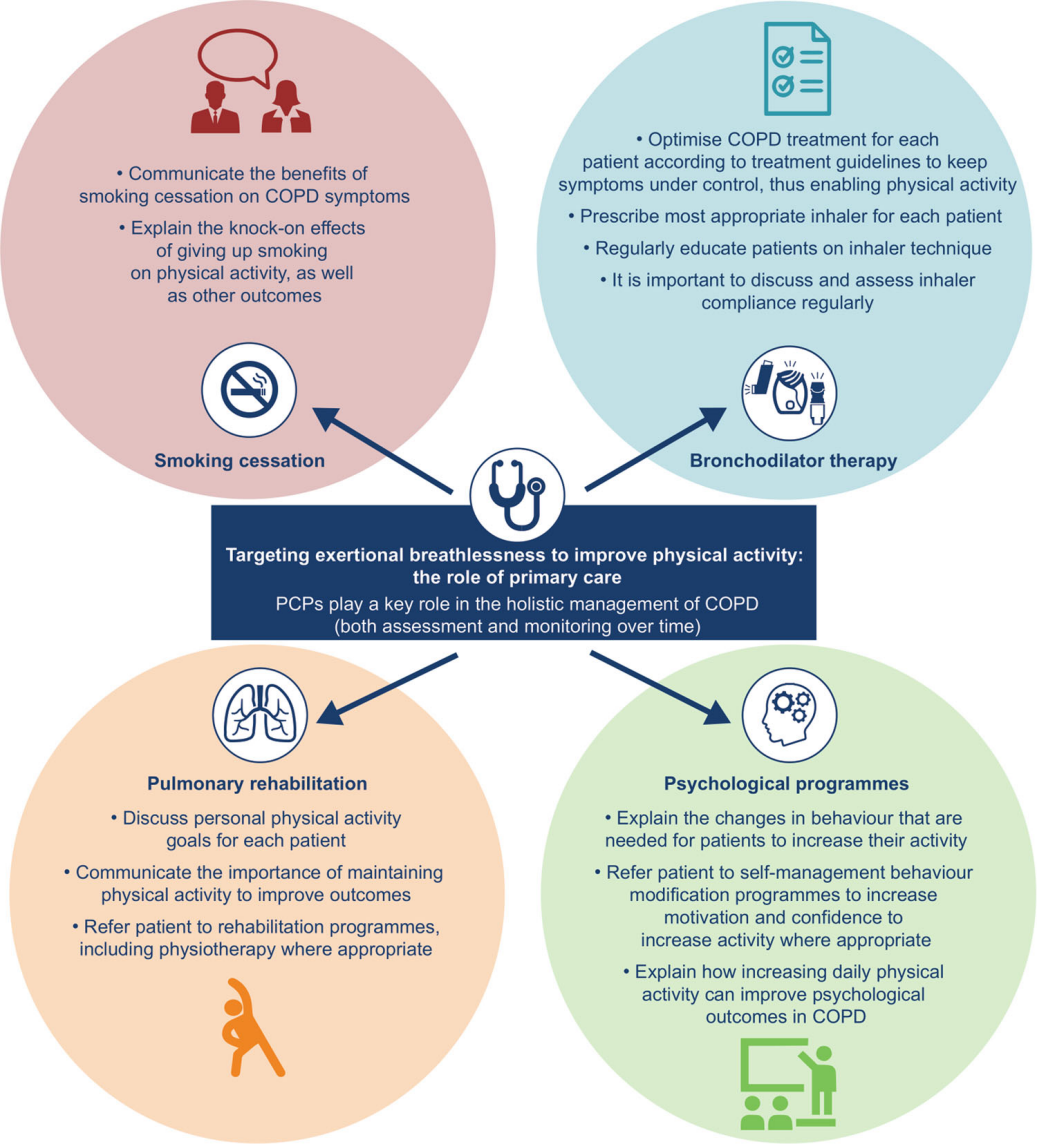
Targeting exertional breathlessness to improve physical activity: the role of primary care. COPD chronic obstructive pulmonary disease, PCP primary care physician.

Increasing physical activity and reducing discomfort during physical activity requires an holistic approach. In addition to the pharmacological approaches discussed above, this should include smoking cessation programmes, pulmonary rehabilitation and psychological programmes^74,75^. Non-pharmacological treatment should consider all aspects of the disease, including mental, physical and emotional health, as well as social implications. Pulmonary rehabilitation is a comprehensive, evidence-based, low-cost treatment intervention that has been proven to improve breathlessness, inactivity and quality of life^74,75^. It is recommended for those patients who have persistent symptoms of COPD and reduced physical activity and who have otherwise not improved through optimal medical management^76^. Exercise training offers benefits that are complementary to the effects gained from pharmacotherapy. Together, pharmacotherapy and rehabilitation enhance health-related quality of life and exercise tolerance and reduce exacerbation rates in patients with COPD^77^. In addition, improving physical activity and exercise tolerance calls for a multidisciplinary approach that is patient-centred. Involving physiotherapy within a patient’s treatment plan has shown positive effects on physical tolerance and levels of dyspnoea^78^. However, access to pulmonary rehabilitation can also be an issue, suggesting the need for restructuring of healthcare resources for COPD in the primary care setting^79^ (Fig. 2).

In addition to the improvements provided by pulmonary rehabilitation and exercise programmes, psychological programmes support the change in behaviour that is needed for patients to increase their activity^80^. For pulmonary rehabilitation and psychological programmes to be successful, PCPs should first ensure that pharmacological management is optimised according to treatment guidelines as an initial step^1,71,72^. In one study, a combination of self-management behaviour modification (SMBM) and tiotropium/olodaterol, with or without exercise training, significantly improved exercise endurance time and breathlessness during daily life in patients with COPD compared with SMBM plus placebo^81^. Physical inactivity in COPD can be affected not only by lung function but also by extra-pulmonary comorbidities^82^. Therefore, effective management of comorbidities is also crucial to help maintain patients’ activity levels.

## Role of PCPs

PCPs have a key role in the diagnosis and management of patients with COPD^83,84^. Encouragement of smoking cessation is a primary goal; it is also important to ensure that influenza vaccinations are kept up to date^84^. Since the vast majority of patients with COPD may suffer from breathlessness on exertion^85^, regular treatment with dual bronchodilators is important to relieve symptoms and improve exercise capacity^71^. PCPs should also support their patients in maintaining or increasing physical activity, which is essential for patient quality of life, ensuring patients understand that their breathlessness, though it may feel debilitating, can be treated. PCPs should encourage their patients to attend pulmonary rehabilitation and should try to help in overcoming any barriers in their participation. Additionally, PCPs need to ensure education on inhaler technique is given at regular intervals during the patient’s follow-up to maintain an optimal treatment strategy. To increase inhaler adherence, it is important to discuss and assess this regularly with the patient.

As PCPs may be responsible for measuring and monitoring patients’ functional status, it is important to consider what is feasible in the primary care setting^46^. There are several tools that are available, each with their own benefits and drawbacks. Although the 6-min walking test is the most reliable test to measure exercise capacity in COPD, it is not very practical for the primary care setting^46^. The physical activity questionnaires presented in the previous section can also be useful, especially the more COPD-specific ones. Indeed, sustained improvements in health status (measured using the CCQ) and exercise capacity have been shown in the primary care setting over 2 years in patients with COPD, emphasising the importance of regular monitoring and follow-up by PCPs to optimise outcomes^86^.

PCPs should discuss personal physical activity goals with their patients, as well as different approaches to meet these goals. Studies have shown that doing activities that are enjoyable promotes physical activity in daily life, suggesting that activities that boost motivation may help patients with COPD to stay active^13^. Strategies may include identifying which activities patients can undertake in the home and discussing options for individuals without access to exercise equipment (e.g. use of water bottles instead of weights). For example, home-based breathing exercises, such as diaphragmatic breathing, yoga breathing, breathing gymnastics and singing, can have significant effects on pulmonary function, respiratory muscle strength, exercise capacity, breathlessness and health-related quality of life in patients with COPD^87^. The ATS/European Respiratory Society official statement on pulmonary rehabilitation reinforces that home-based exercises have been proven to effectively reduce dyspnoea and increase exercise performance in patients with COPD^88^. In addition, some patients may be considered for referral to multicomponent rehabilitation programmes. The patient should be made aware of anything their local communities are setting up, such as programmes with physiotherapists and walking groups, to incorporate social interaction into their rehabilitation.

## Conclusions

The complexity and heterogeneity of COPD make it a challenging disease to treat, and patients’ avoidance of activity, and subsequent decline in capacity to perform, further impact the management of the disease. Improving patient tolerance of physical activity, increasing participation in daily activities and helping patients to remain active are clear goals of COPD management. These require an holistic approach to management, including smoking cessation, pulmonary rehabilitation and psychological programmes in parallel with bronchodilator therapy, in order to address both physiological and behavioural factors. PCPs have an important role to play in optimising therapy, setting goals and relaying the importance of maintaining physical activity to their patients. In addition, optimal treatment that addresses activity-related breathlessness can help prevent the downward spiral of inactivity and get patients moving again to improve their overall health and long-term prognosis.
